# Supplementary material for: Realizing Both Antibacterial Activity and Cytocompatibility in Silicocarnotite Bioceramic via Germanium Incorporation
Source: J Funct Biomater. 2023 Mar 14;14(3):154. doi: 10.3390/jfb14030154 (PMC10054726; doi:10.3390/jfb14030154)
Supplement: Supplementary file 1 [file jfb-14-00154-s001.zip › jfb-2188178-supplementary.pdf]

Journal of
Functional
Biomaterials

MDPI

Article

Supplementary Materials: Realizing Both Antibacterial Activity and Cytocompatibility in Silicocarnotite Bioceramic via Germanium Incorporation

Yingqi Ji [1,2], Shun Yang [1,2], Jian Sun [3,*] and Congqin Ning [4,*]

[1] State Key Laboratory of High Performance Ceramics and Superfine Microstructure, Shanghai Institute of Ceramics, Chinese Academy of Sciences, Shanghai 200050, China
[2] Center of Materials Science and Optoelectronics Engineering, University of Chinese Academy of Sciences, Beijing 100049, China
[3] Department of Prosthodontics, Shanghai Ninth People's Hospital, Shanghai Jiao Tong University School of Medicine, Shanghai 200011, China
[4] The Education Ministry Key Lab of Resource Chemistry, Shanghai Key Laboratory of Rare Earth Functional Materials and Shanghai Frontiers Science Center of Biomimetic Catalysis, Shanghai Normal University, Shanghai 200234, China
* Correspondence: doctorsunjian74@163.com (J.S.); cqning@shnu.edu.cn (C.N.)

Inhibition diameter assessment

In brief, 100 μL bacteria suspension (10^7 cfu/mL) including *S. aureus* or *E. coli* was seeded on TSB or LB agar medium rspectively, and the sample was put on its surface and then cultured for 18 h at 37 °C in an incubator under the atmosphere. The antibacterial ability was investigated by the area of inhibition rings around the specimens (the bigger the area, the better the antibacterial property).

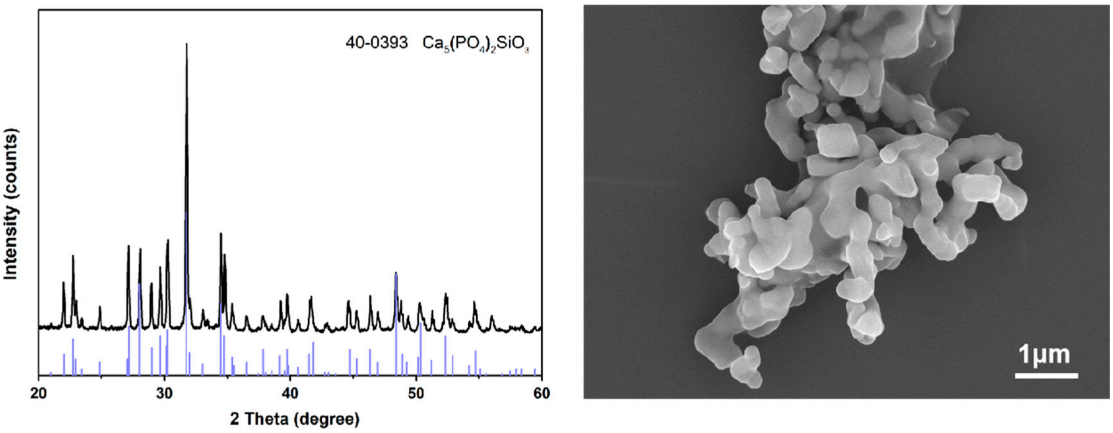

Figure S1. XRD patterns and SEM morphologies of the CPS powders.

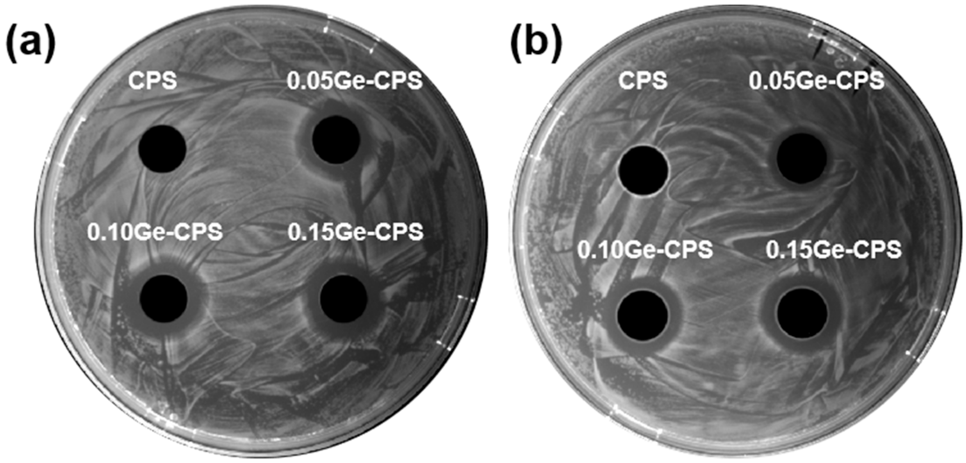

Figure S2. Inhibition rings around CPS, 0.05Ge-CPS, 0.10Ge-CPS and 0.15Ge-CPS samples against (**a**) *E. coli* and (**b**) *S. aureus.*
